# Bromelain in Obesity Therapy: A Review of Anti-Inflammatory and Metabolic Mechanisms

**DOI:** 10.3390/ijms26178347

**Published:** 2025-08-28

**Authors:** Yashvi Sethia, Ewelina Polak-Szczybyło, Jacek Tabarkiewicz

**Affiliations:** 1 Students’ Scientific Club, Department of Human Immunology, Faculty of Medicine, Medical College of Rzeszow University, University of Rzeszow, 35-959 Rzeszow, Poland; ys128350@stud.ur.edu.pl; 2 Department of Dietetics, Faculty of Health Sciences and Psychology, Collegium Medicum, University of Rzeszow, 35-959 Rzeszow, Poland; ewpolak@ur.edu.pl; 3 Department of Human Immunology, Faculty of Medicine, Collegium Medicum, University of Rzeszow, 35-959 Rzeszow, Poland; 4 Laboratory for Translational Research in Medicine, Centre for Innovative Research in Medical and Natural Sciences, Medical College of Rzeszow University, University of Rzeszow, 35-959 Rzeszow, Poland

**Keywords:** bromelain, obesity, anti-inflammatory, immunomodulatory, phytotherapeutic, chronic low-grade inflammation, cardiovascular diseases, diabetes

## Abstract

The increasing prevalence of obesity, a chronic disease, necessitates the development and evaluation of evidence-based prevention and intervention strategies tailored to heterogeneous populations. Certain fruits, including papaya and pineapple (*Ananas comosus*), have been investigated as potential dietary components in obesity management. In the context of obesity and chronic low-grade inflammation, bromelain, a proteolytic enzyme derived from pineapple, is a widely studied phytotherapeutic agent that acts through multiple mechanisms intersecting immune and metabolic pathways. This narrative review summarizes current evidence on the effects of bromelain in obesity, low-grade inflammation, and related metabolic disturbances. Searches of the literature were conducted in Google Scholar, PubMed, and Scopus databases. This review incorporates findings from in vitro, animal, and human studies. We outline the mechanisms and evidence supporting the therapeutic efficacy of bromelain, emphasizing its implications for obesity management in clinical settings. Bromelain has been shown to exert significant anti-inflammatory activity and may modulate adipocyte metabolism, potentially alleviating comorbidities associated with excess adiposity. Although its effects on immune cells are relatively well described, the mechanisms underlying bromelain’s actions on adipocytes remain incompletely understood.

## 1. Introduction

The global obesity epidemic represents a multifaceted public health challenge with prevalence rates having doubled in over 70 countries since 1980 [1]. Obesity, recognized as a chronic and complex disease, requires urgent interventions to curb the crisis. In 2022, it was estimated that 1 in 8 people in the world were living with obesity along with 37 million children under the age of 5. Obesity is no longer confined to high-income countries but has also shown a steady increase in low- and middle-income regions [2]. The global prevalence of obesity is estimated to rise to over 50% of the world’s population by 2035. It has been reported that this rise in obesity is expected to be steepest among children and adolescents, rising from 10% to 20% [3].

Numerous studies and reports have highlighted the need to consider and find possible ways to manage the epidemic of obesity. Currently, numerous conventional pharmacological agents are employed in the therapeutic management of obesity; however, their limited accessibility and associated adverse effects constrain their widespread use. Consequently, there exists a critical demand for the development of safe, efficacious, economically viable, and readily accessible therapeutic alternatives [4]. In recent years, there has been a marked surge in interest regarding the medicinal potential of plants and the application of naturally derived phytochemicals. Plants are increasingly recognized as integral components of the human diet and as critical reservoirs of natural bioactive compounds possessing significant biological activity [5].

Bromelain, a principal sulfhydryl-dependent proteolytic enzyme extracted from *Ananas comosus*, constitutes a highly promising phytotherapeutic agent with extensive utility across multiple branches of modern medicine. *Ananas comosus*, a member of the *Bromeliaceae* family and one of the most extensively consumed tropical fruits, is widely cultivated across a range of tropical and subtropical regions, including but not limited to Thailand, Indonesia, Malaysia, India, Kenya, China, and the Philippines. Bromelain, a cysteine protease that catalyzes proteolytic processes, has been shown to remain stable for extended periods at temperatures below 20 °C [6].

All structural components of the pineapple plant—including the stem, core, peel, crown, and leaves—can be utilized for the extraction of bromelain, although notable differences exist in the concentrations and compositional profiles. The stem and fruit produce markedly higher yields of bromelain compared to the core, peel, and leaves; collectively, the stem, crown, and other parts contribute approximately 50% (*w*/*w*) of total pineapple waste, thereby making bromelain recovery both economically viable and ecologically sustainable. As a result, bromelain isolated from the stem is regarded as the most practical and therapeutically potent form, demonstrating enhanced proteolytic activity relative to its fruit-derived counterpart [5]. The bromelain extract, in addition to various thiol endopeptidases, also includes other components such as phosphatases, glucosidases, cellulases, peroxidases, glycoproteins, carbohydrates, several protease inhibitors, and organically bound Ca^2+^. It is assumed that the percentage composition of the bromelain extract consists of 80% stem bromelain, 10% fruit bromelain, 5% ananain, and other ingredients [5,6]. One of the primary challenges associated with the use of enzymes, such as bromelain, is the decline in their biological activity following processing or during storage over time. Immobilization has emerged as a promising strategy to maintain enzymatic functionality. Various immobilization techniques have been explored for bromelain, including entrapment within hydrogels, adsorption onto chitosan matrices, covalent attachment, and encapsulation within nanoparticles, which has recently demonstrated sustainable results, thereby enhancing the anti-proliferative and antioxidant activities of bromelain in a manner dependent on the duration of exposure [6]. Absorption of bromelain occurs mainly through the digestive tract. It is estimated that approximately 40% of functionally intact bromelain is present in the blood of rats after oral administration of the extract, with the highest concentration observed up to an hour after administration, having a half-life of 6–9 h [7]. The efficient absorption of bromelain occurs due to its capacity to bind to the two main blood antiproteases, alpha1-antichymotrypsin and alpha 2-macroglobulin, which stabilize even dilute solutions of bromelain [5,7].

**Figure 1:**
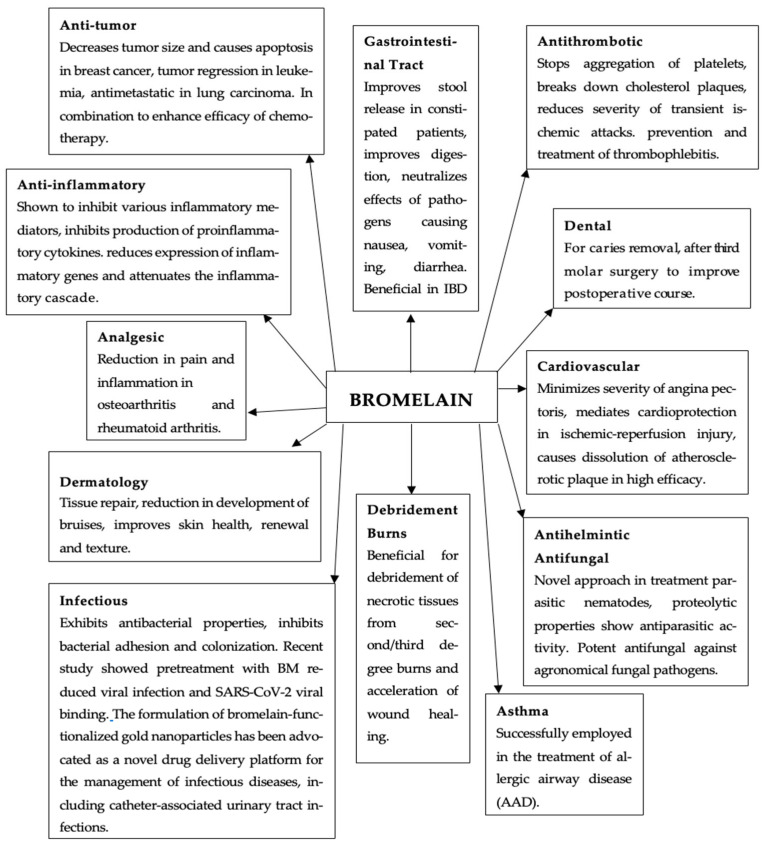
Therapeutic potential of bromelain [5,7,8,9,10,11,12,13,14,15,16,17].

Bromelain is generally regarded as a safe nutraceutical with potential effectiveness, has been applied in addressing a variety of health disorders, and is widely available as a dietary supplement with reported health benefits. Its biological activity continues to be the subject of extensive scientific investigation. It has been increasingly applied across multiple industries, including cosmetology, pharmaceuticals, food production, and biotechnology. Moreover, accumulating scientific evidence suggests that bromelain extracts may possess antitumor properties and could potentially enhance the efficacy of certain chemotherapeutic agents [8]. The diverse therapeutic applications of bromelain are summarized in Figure 1.

By providing favorable conditions for enzymatic activity, bromelain may enhance overall digestive efficiency. Moreover, its anti-inflammatory properties could confer cytoprotective effects on pancreatic and intestinal tissues, thereby maintaining tissue integrity and preserving their capacity to secrete digestive enzymes effectively [17].

Inflammation is an integral component of obesity-associated diseases. Obesity promotes a pro-inflammatory environment by elevating the levels of inflammatory mediators such as IL-6 (interleukin) and tumor necrosis factor alpha (TNF-α), while decreasing adiponectin, a molecule that has anti-inflammatory properties. The upregulation of pro-inflammatory cytokines in obesity is regarded as a key mediator linking excess adiposity with systemic inflammation. The relationship between inflammatory markers and obesity measurements in individuals with normal and high blood pressure was examined. In obese hypertensive individuals, the waist-to-height ratio (WHR), an effective measure of visceral fat, was linked to chronic inflammation. Additionally, the body adiposity index (BAI) showed a correlation with C-reactive protein (CRP) levels, independent of both hypertension and gender [18]. In the liver, adiponectin improves insulin sensitivity, decreases uptake of non-esterified fatty acids, reduces gluconeogenesis, and increases the oxidation process. Since low levels of adiponectin are consequent to obesity, it is shown to be one of the mechanisms of development of insulin resistance in obese patients. The increased levels of free fatty acids (FFAs) found in obese individuals also contribute to the defects in glucose use and storage. Moreover, obesity is correlated with various post-receptor binding defects in insulin signaling, including compromised generation of second messengers, impaired glucose transport, and disturbances in essential enzymatic pathways involved in glucose utilization [19]. A marked increase in fasting glucose and insulin levels after 11 weeks of high-fat feeding was demonstrated in a study on a mouse obesity model. The fasting glucose to insulin ratio is commonly used as an indicator of insulin resistance, with values below 4–5 often considered abnormal in humans. In this study involving mice, the subjects developed obesity and exhibited typical signs of insulin resistance [20].

As there is an established link between obesity, inflammation, and insulin resistance which then further exacerbates the patient’s condition, this review highlights the potential role of bromelain in acting as an anti-inflammatory and thereby in potential obesity therapy. Despite considerable progress in understanding the etiology and management of obesity, the extensive rise in its prevalence highlights the lack of effective large-scale clinical interventions [21]. The major consequences of obesity are summarized in Table 1.

In this review, we comprehensively examine the diverse mechanisms and supporting evidence through which bromelain exerts its therapeutic effects, substantiating its potential role in the management of obesity. Given the multifaceted properties of bromelain and the relatively underexplored adipocyte-related mechanism in the context of inflammation and obesity, this review aims to synthesize findings across multiple studies and establish its relevance in potential adjunctive therapy.

## 2. Mechanisms of Bromelain Action in Obesity and Inflammation

### 2.1. Bromelain as a Regulator of Adipocyte Function and Metabolic Signaling

Considering the molecular mechanisms of bromelain’s effect on adipogenesis, apoptosis, and lipolysis, it should be mentioned that adipogenesis is controlled, among others, by a transcriptional cascade involving C/EBP (CCAAT/enhancer-binding protein) and PPARγ (PPARc peroxisome proliferator-activated receptor gamma) proteins. C/EBPβ and C/EBPδ are rapidly and transiently activated as a result of differentiation signals, while C/EBPβ is essential for mitotic clonal expansion (MCL), which is involved in adipocyte differentiation. Both previously mentioned proteins are also essential for adipocyte differentiation [41,42]. Exposure of preadipocytes to bromelain leads to a decrease in the mRNA (messenger ribonucleic acid) level of C/EBPα and PPARγ, but it has no effect on C/EBPβ and C/EBPδ [43]. Bromelain also may affect preadipocyte differentiation by affecting the level of adiponectin by reducing its expression and secretion [43]. It is shown that the Akt–TSC2–mTORC1 (protein kinase B–tuberous sclerosis complex 2–mechanistic target of rapamycin complex 1) pathway stimulates PPARc expression and adipogenesis [44]. Bromelain has been shown to inhibit the expression of PPARc target genes ap2 (adipocyte protein 2 also known as FABP4—fatty acid binding protein), FAS (fatty acid synthase), LPL (lipoprotein lipase), CD36 (cluster of differentiation), and ACC (acetyl-CoA carboxylase). Additionally, bromelain may affect the phosphorylation of Akt in adipocytes, which promotes their apoptosis and reduces their viability [43]. Adipocyte apoptosis is an important element of the mechanism of reducing the volume of adipose tissue and thus obesity [45]. Bromelain may also interfere with this mechanism by modifying the TNF-α pathway in adipocytes, which is also associated with the selective removal of adipocytes, but not preadipocytes [45]. TNF-α may disrupt the normal regulation of energy metabolism, and its overexpression contributes to the reduction in the number of adipocytes via apoptosis and inhibition of insulin action, while TNF-α-induced lipolysis reduces the expression of the anti-lipolytic genes PDE3B (phosphodiesterase 3B) and Gia1 (inhibitory G protein alpha subunit 1) [46].

Bromelain has been reported to reduce the levels of adipose tissue-derived cytokines (e.g., TNF-α) that lead to serine phosphorylation of the IRS-1 (insulin receptor substrate 1) protein. This unblocks insulin signaling into the cell, especially in muscle and adipocytes. This mechanism may improve glucose metabolism and reduce insulin resistance [47]. TNF-α is one of the key cytokines inhibiting insulin signaling by phosphorylating IRS-1 (insulin receptor substrate 1) on serine residues (inhibitory) and inhibiting GLUT4 (glucose transporter type 4) translocation. Bromelain may affect the level of TNF-α—both increasing it in adipocytes and decreasing it in other inflammatory contexts (e.g., in the intestine, liver), which may be dose- and model-dependent [43].

Animal studies have shown that bromelain may reduce macrophage infiltration into adipose tissue and shift the phenotype of macrophages from pro-inflammatory M1 (classically activated macrophages) to anti-inflammatory M2 (alternatively activated macrophages). The M2 macrophage phenotype improves the metabolic homeostasis of adipocytes and reduces oxidative stress and restores insulin sensitivity [48]. There is no direct data on the effect of bromelain on the expression of the leptin (EP) gene, but decreased M1 activity may also affect the reduction in leptin levels. Studies on humans with obesity and on an animal model have shown a reduction in leptin in the group consuming pineapple juice, which is a source of bromelain [49,50].

Bromelain may reduce lipopolysaccharide (LPS) translocation (metabolic endotoxemia), a known factor contributing to inflammation in obesity, and penetration of LPS from the intestinal microbiota plays an important role in the exacerbation of obesity and its metabolic complications. Among other things, it activates the immune system through the TLR4 receptor (Toll-like receptor 4); stimulates the production of the inflammatory cytokines TNF-α, IL-6, and IL-1β (interleukin 1 beta); and induces low-grade inflammation. Additionally, pro-inflammatory cytokines activated by LPS disrupt signaling through the insulin receptor (IRS-1) and cause glucose uptake disorders due to impaired GLUT4 translocation. Indirectly, in obesity, LPS promotes adipocyte enlargement and inflammation [51,52].

Traditionally, bromelain has been used to support protein digestion and gastrointestinal function. There are several aspects to this. The proteolytic activity of bromelain helps break down proteins into peptides and amino acids, which may affect satiety and energy metabolism. Supporting protein digestion promotes a favorable environment for enzymatic activity and increases overall digestive capacity and nutrient absorption, which also affects the microbiome [17].

### 2.2. The Effect of Bromelain on the Immune System and Inflammatory Pathways

Bromelain affects the immune system through multiple mechanisms. Among others, bromelain modifies the regulation of pro-inflammatory and anti-inflammatory cytokines. This occurs by inhibiting the activation of the transcription factor NF-κB, which reduces the expression of inflammatory genes and weakens the inflammatory cascade [53].

Bromelain also affects the modulation of surface adhesion molecules on the surface of T lymphocytes and NK (natural killer cells) and macrophages. In studies using LPS, bromelain significantly decreased ICAM-1 (intercellular cell adhesion molecule 1), and VCAM-1 (vascular cell adhesion molecule 1) [54]. ICAM-1- and VCAM-1-positive cells play a significant role in inflammatory processes by activating T cells, participating in the adhesion of leukocytes to endothelial cells, and helping to recruit leukocytes to inflamed areas [55]. In addition, bromelain suppresses signaling pathways essential for immune response such as the nuclear factor kappa B (NF-κB) pathway and the Raf-1/extracellular regulated kinase (ERK-) 2 pathway. In this way, bromelain reduces the expression of inflammatory genes and attenuates the inflammatory cascade and disrupts the signaling processes necessary for T cell activation [53]. By reducing the activity of the NF-κB and MAPK (mitogen-activated protein kinase) pathways, it affects the reduction in inflammatory mediators [56]. Inhibition of the MAPK pathways, in studies, results in a decrease in the level of cyclooxygenase-2 (COX-2), PGE2 (prostaglandin E2) mRNA, s and c-Jun N-terminal kinase (JNK). It regulates the activation of p38 and inhibits the phosphorylation of c-Jun and c-Fos [57]. These proteins are part of the AP-1 complex (activator protein 1; transcription factor complex including c-Jun and c-Fos), which is significantly associated with cell proliferation, apoptosis, and transformation [58]. Bromelain in macrophages inhibits the activity of macrophage inflammatory protein 1 alpha (MIP-1α), macrophage inflammatory protein-1 beta (MIP-1β), and monocyte chemotactic protein-1 (MCP-1) [59,60,61].

Bromelain additionally inhibits the expression of iNOS (inducible nitric oxide synthase) mRNA while limiting oxidative stress. The iNOS enzyme synthesizes nitric oxide (NO), which is also a pro-inflammatory factor [62]. Bromelain has been shown in vitro to remove cell surface receptors CD128a/CXCR1 (chemokine receptor 1) and CD128b/CXCR2 (chemokine receptor 2), responsible for (interleukin-8) IL-8-induced neutrophil migration. Studies have shown a 40% decrease in neutrophil migration in human leukocytes treated with bromelain [63]. In the study by Hale et al., 14 of the 59 surface markers analyzed were classified as bromelain-sensitive and depleted (CD7, CD8α, CD14, CD16, CD21, CD41, CD42a, CD44, CD45RA, CD48, CD57, CD62L, CD128a, CD128b), which are associated with anti-inflammatory properties. Others showed only partial sensitivity (CD4, CD40, CD56, CD61, CD79, CD132). Additionally, it was shown that bromelain increased the reactivity of surface markers by exposing epitopes as a result of proteolysis (CD5, CD11b, CD11c, CD13, CD15, CD18, CD53). Removal of surface molecules reduces the ability of leukocytes to migrate, hinders their activation, and weakens communication between cells of the immune system, which affects the suppression of the inflammatory response [64].

### 2.3. The Role of Bromelain in Obesity-Related Low-Grade Inflammation

Low-grade inflammation is a condition that combines both obesity and chronic inflammation. The common mechanisms that bromelain can interfere with are the reduction in pro-inflammatory cytokines (TNF-α, IL-1β, IL-6), which are excessively produced both in obesity (by macrophages in adipose tissue) and in other inflammatory conditions. Bromelain has demonstrated the ability to suppress them in both contexts. This substance alleviates oxidative stress by supporting cellular antioxidant mechanisms [65]. It should be noted that oxidative processes are intensified in obesity, and oxidative stress in individuals with excess body weight is one of the causes of many diseases [66]. Bromelain organically influences the effects of dysbiosis and LPS translocation occurring in obesity [51,52,67].

**Figure 2:**
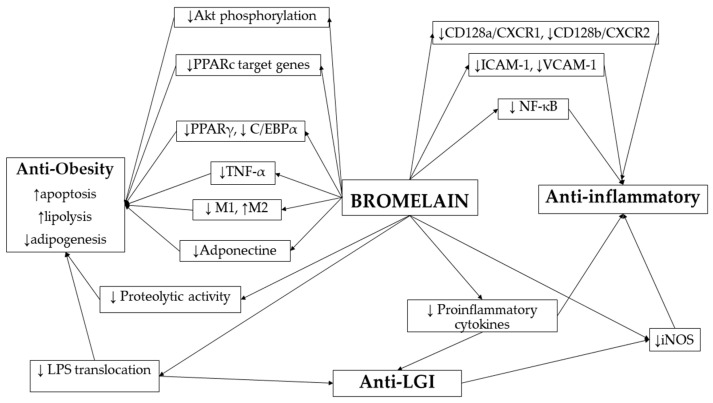
Bromelain signaling pathways during anti-obesity, anti-inflammation, and anti-low-grade-inflammation effects [41,42,43,44,45,46,47,48,49,50,51,52,53,54,55,56,57,58,59,60,61,62,63,64,65,66,67]. The effects of bromelain are marked as follows: ↑ increased, ↓ decreased.

Figure 2 summarizes bromelain signaling pathways in the context of anti-obesity, anti-inflammatory, and anti-low-grade-inflammation effects. Bromelain appears to exert multifaceted biological effects by modulating key molecular targets involved in adipogenesis (↓PPARγ, ↓C/EBPα), lipolysis, and apoptosis (↑Akt phosphorylation, ↓adiponectin), as well as immune signaling (↓TNF-α, ↓NF-κB, ↓iNOS, ↓pro-inflammatory cytokines). It influences macrophage polarization (↓M1, ↑M2), surface adhesion molecules (↓ICAM-1, ↓VCAM-1), and chemokine receptors (↓CD128a/b). These mechanisms are interrelated but partially independent, which contributes to the potential therapeutic role of bromelain in metabolic and inflammatory disorders and the treatment of obesity.

### 2.4. Potential Effect of Bromelain on Circadian Regulation of Adipose Tissue

In addition to lifestyle factors, adipose tissue activity is influenced by a highly active endogenous (24-h) clock. This affects both adipocyte progenitor cells and mature adipocytes and may play a unique role in the health and function of adipose tissue. Individual nutrients may also influence the circadian clock. The core molecular clock is involved in the regulation of lipogenesis and lipolysis, as many key enzymes involved in these processes are directly regulated by the CLOCK:BMAL1 transcription complex. Lipolysis in WAT is rhythmic, resulting in the daily release of FFAs and glycerol into the blood [68]. It is important to note that BMAL1 recruitment to target genes in adipose tissue is remodeled under conditions of obesity, and this remodeling can be reversed by inhibiting nuclear factor kappa B (NF-κB) [69]. Although there are no direct studies on the effect of bromelain on the activity of genes responsible for circadian rhythm in adipose tissue, bromelain inhibits NF-κB, which may reverse the negative effects of circadian clock disruption in obesity and adipocyte proliferation. Furthermore, the biological clock influences the secretion of adipokines, the levels of which can also be reduced by bromelain. In mice with a global Clock gene knockout, leptin production is increased and adiponectin is decreased [70]. Polyphenols such as resveratrol, proanthocyanidins, epigallocatechin gallate, and nobiletin have been shown to modulate the expression of core clock and clock-controlled genes while simultaneously reducing adipose tissue by decreasing adipocyte volume [71,72,73]. Other ingredients include alkaloids such as caffeine, which lengthens the period of BMAL1 expression in NIH3T3 cells [74]. Unfortunately, the effects of proteolytic enzymes such as bromelain remain untested.

## 3. Evidence from In Vitro (Cell) and In Vivo (Human, Animal) Studies Related to the Functions of Bromelain—Immunomodulation, Fibrinolysis, Treatment of Obesity, Inflammation, Cardiovascular Diseases, NAFLD, and Diabetes

Bromelain acts at the intersection of innate immunity, metabolic processes, and inflammatory pathways, and may be considered a potential supportive strategy for inflammatory-metabolic diseases such as obesity, cardiovascular disease, diabetes, and NAFLD. The potential efficacy of bromelain, as demonstrated in various studies, is discussed below. Particular attention is given to studies related to obesity, its impact on the immune system, and associated metabolic disorders.

### 3.1. Obesity-Related Models

Research on obesity-related parameters often utilizes pineapple juice, which complicates the attribution of observed effects specifically to bromelain, as the juice also contains vitamins, minerals, and polyphenols. These studies are also analyzed in the context of obesity, due to the importance of the topic of the review.

In a randomized, single-blind, controlled trial on 52 obese patients with diabetes type 2, it was shown that after 8 weeks of supplementation with 500 mg of bromelain twice a day, the BMI and waist and hip circumferences decreased compared to the baseline [50]. However, in the study by Ley et al. among 68 diabetic patients, supplementation did not result in any changes in body weight, BMI, and waist and hip circumferences [75].

In a study on mice by Liu et al., they found that bromelain fibrils showed significantly stronger interfacial stabilization of oil-in-water emulsions than papain fibrils, which resulted in significantly higher lipid content in the feces. This phenomenon may have a protective effect in a high-fat diet (HFD) and prevent weight gain [76]. In another animal study, body weight gain was also induced by an HFD administered for 12 weeks. Then, mice were divided into two groups, where in one group the food was supplemented with bromelain (20 mg/kg). In the bromelain group, body mass was reduced by about 30%, liver mass by about 20%, and white adipose tissue mass by about 40%. In addition, lipid accumulation in the liver was observed in this group. The authors of the study emphasized that the effect of bromelain, a proteolytic enzyme derived from pineapple, may consist of the reduced uptake of free fatty acids and reduced synthesis of cholesterol esters as well as increased internalization of lipoproteins. Bromelain may also affect the metabolism of bile acids and the regulation of the expression of proteins involved in numerous metabolic pathways in the liver [77,78]. These findings suggest a potential role of bromelain in the treatment of obesity but also in metabolic syndrome. It is also reported that bromelain supplementation in an animal model reduced body weight, decreased the amount of WAT (white adipose tissue), increased the mass of BAT (brown adipose tissue), and reduced the size of WAT adipocytes and BAT lipid droplets [54].

Studies related to obesity and the effect of bromelain on adipocyte activity or body weight reduction are insignificant. In an in vitro study on preadipose 3T3 clonal cell line (3T3-L1—preadipocyte cell line derived from mouse embryonic fibroblasts) cells, it was shown that stem bromelain (SBM) inhibited their differentiation by reducing adipogenic gene expression and induced apoptosis and lipolysis in mature adipocytes. Decreased mRNA levels of adipocyte fatty acid-binding protein, FAS, LPL, CD36, and ACC were also noted [43].

The main effects in the context of bromelain in obesity are summarized in Table 2.

### 3.2. Anti-Inflammation and Immunomodulation

Bromelain exhibits anti-inflammatory properties, but in various contexts it can also modulate inflammation. This is usually dependent on variables such as dose, model, duration of application, and formulation. Low to moderate doses (250–2000 mg) administered orally have been shown to reduce levels of IL-6, TNF-α, COX-2, and NF-κB, resulting in anti-inflammatory effects, but very high doses can promote immune activation in some models [79]. Several in vitro studies demonstrate increased levels of cytokines such as IL-6, TNF-α, and IFN-γ by macrophages and lymphocyte cultures after bromelain supplementation [80,81,82].

The immunomodulatory and anti-inflammatory properties of bromelain have been studied for their efficacy in various inflammatory diseases. Scientific studies supporting its properties are discussed below. In vitro studies on intestinal biopsies from patients with inflammatory bowel diseases (IBDs) showed that the addition of bromelain modified the activity of G-CSF (granulocyte-stimulating factor), reducing the secretion of IFN-γ (interferon gamma), CCL4/macrophage inhibitory protein (MIP)-1β, and TNF-α. Animal studies also suggest beneficial effects of bromelain in the treatment of indomethacin-induced IBD. Bromelain was used in rats for 3 weeks at a dose of 1000 mg/kg/day. The level of TNF-α and IL-10 (interleukin 10) was effectively reduced, but not below the physiological value, because it was excessively elevated before exposure to bromelain. Additionally, MCP-1, PGE2, NF-κB, CRP (C-reactive protein), and MPO were reduced [83]. In the study by Zhou et al., the effect of bromelain was analyzed in a model of colitis in rats induced by enteral administration of 2,4,6-trinitrobenzosulfonate (TNBS) and in the intestinal epithelial cells of rats (IEC-6) and humans (Caco-2) using LPS. In vivo, bromelain was reported to alleviate symptoms of inflammation (dose-dependent), restore intestinal barrier function (↓ FD-4, ↑ occludin, ↓ MLCK) (myosin light chain kinase), and inhibit the expression of TNFRs, NF-κB, MLCK, and apoptosis markers [84]. Also, in another study on an animal model, after induction of intestinal inflammation by doses of indomethacin and then after 3 weeks of treatment with bromelain at a dose of 1000 mg/kg/day, blood and tissue were collected. In the blood, the level of IL-10 was reduced, and TNF-α, MCP-1, CRP, NF-κB, and PGE2 were reduced. In intestinal biopsies, it significantly reduced oxidative stress [83]. The anti-inflammatory effect of this substance may also alleviate intestinal inflammation induced by gliadin in individuals with celiac disease. It effectively degrades gliadin and modulates the immune system [85].

Bromelain’s properties can be used in the treatment of inflammation of the gums and dental pulp. It has anti-inflammatory and antioxidant effects in periodontal diseases, and it appears to reduce inflammation by reducing the amount of cytokines. Lee et al., in a study on RAW 264.7 mouse macrophages, showed that it has anti-inflammatory and antioxidant effects and can be an effective tool in the treatment of inflammation of the gums. Its mechanism of action includes inhibition of the iNOS, COX-2, and MAPK and AP-1 pathways [56]. Bromelain (100 µg/mL) significantly inhibits inflammatory responses. Human gingival fibroblasts (HGFs) were exposed to AGEs (advanced glycation end-products) with or without bromelain at concentrations ranging from 2.5 to 40 µg/mL. Doses up to 20 µg/mL after 24 h inhibited the production of IL-6 and IL-8 and the expression of the aging marker p16. It also inhibited the activation of p65, p-ERK, and p-p38 proteins. The mechanism of action, according to the researchers, includes the inhibition of the NF-κB and MAPK/ERK [51]. Bromelain appeared to improve the periodontal condition; reduce the number of leukocytes; improve bone integration; inhibit bone resorption; reduce the levels of IL-6, TNF-α, M-CSF (macrophage colony-stimulating factor), RANKL (receptor activator of nuclear factor κ B ligand), and MMP-8 (matrix metalloproteinases); increase the levels of osteoprotegerin (OPG), GPx, and SOD; and reduce MDA [86], and this was confirmed in a human model by inhibiting the activation of the NF-kB and MAPK/ERK pathways [65]. Bromelain not only has an anti-inflammatory effect but also an effect in supporting the regeneration of dental pulp. In a study on human dental pulp cells isolated from children’s supernumerary teeth, inflammation was simulated using LPS and the effect of bromelain at different concentrations of 2.5, 5, 10, 20, and 40 µg/mL was examined. Inhibition of the expression of cytokines IL-1β, IL-6, and IL-8 (mRNA + protein); adhesion factors ICAM-1 and VCAM-1; as well as signaling pathways ERK, p38 MAPK, and NF-κB (p65) was demonstrated. After 7 and 14 days, the effect of pulp mineralization was also discovered [56].

Anti-inflammatory properties have been demonstrated in many other studies. A model of chronic sciatic nerve injury (CCI) was induced in Wistar rats. After 21 days of treatment, bromelain significantly reduced pain symptoms. The levels of IL-1β, IL-6, and PGE2 in the cerebral cortex were reduced, as well as NF-κB, IL-1β, IL-6, TNF-α, PGE2, and nitrates in the sciatic nerve by reducing iNOS expression. Bromelain may suppress not only inflammatory but also nitrosative stress [87]. Histopathological examination of the heart and liver tissues of rats treated with bromelain showed a reduction in the expression of genes responsible for the production of IL-1β, IL-6, and TNF-α [88]. In another in vivo and in vitro study on a mouse model using splenocytes also taken from mice, bromelain was found to increase T cell proliferation following stimulation by TCR and anti-CD28 antibodies in splenocytes cultures by enhancing the costimulatory effects of accessory cells. However, despite increased T cell proliferation, bromelain simultaneously decreased IL-2 production in these cultures. Bromelain did not affect the proliferation of purified CD4^+^ T cells, but inhibited IL-2 production in these cells as well. In vivo, bromelain also enhanced the immune response of lymphocytes [82]. In a study on macrophages differentiated from human U937 monocytes and treated with LPS to activate the inflammatory pathway in macrophages, increasing the expression of cytokines and chemokines, bromelain inhibited the expression of MIP-1α, MIP-1β, MCP-1, IL-8, IL-1β, IL-6, and COX-2 [89]. Bromelain, in a study on osteoarthritis (OA) and rheumatoid arthritis (RA) using porcine cartilage and SW982 synovial fibroblasts, reduced the level of pro-inflammatory cytokines (TNF-α, IL-1β, IL-6, IL-8); inhibited the phosphorylation of p65 (NF-κB), p38, and JNK (MAPK) pathways; and reduced the degradation of GAG, HA, and collagen in cartilage [90].

The anti-inflammatory effect of bromelain is very often dose-dependent. The higher the dose of bromelain, the lower the production of cytokines and pro-inflammatory mediators induced by LPS in RAW 264.7 mouse macrophages, which correlated with a decrease in iNOS and COX-2 expression. Bromelain also inhibited the phosphorylation of p65 (NF-κB), an inhibitor of IκB-α, ERK, JNK, and p38 kinases (p38 MAP kinase—a subfamily of MAPK) in LPS-treated cells. Purified bromelain (PBM) showed a stronger anti-inflammatory effect than crude (CBM) [91]. Also, a study on RAW264.7 mouse macrophages showed an anti-inflammatory effect of bromelain (1–100 microg/mL) by inhibiting the activity of NF-κB but also the phosphorylation of MAPKs (ERK, p38, JNK) as well as the production of nitric oxide (NO) and pro-inflammatory cytokines. In vitro, bromelain decreased the expression of TNFR2, NF-κB, and MLCK in response to LPS, and in Caco-2 PFB, it reversed the decrease in TER (transepithelial electrical resistance) [84]. Another in vitro study also suggested that the potency of bromelain was largely dose-dependent. It stimulated human peripheral blood mononuclear cells (PBMCs) to produce pro-inflammatory cytokines. The observation that TNF-α production appears after 4–6 h, reaches a plateau after 12–16 h, and disappears after 24 h of incubation seems significant [92].

It is worth noting that bromelain may act in a supportive and synergistic manner with other anti-inflammatory substances. Ghensi et al. used mesenchymal cells and applied bromelain, dexamethasone sodium phosphate, and a mixture of both. Both bromelain and dexamethasone reduced inflammation (increased IL-10 levels, decreased IL-1), but the combination of these substances had a stronger anti-inflammatory effect. Both of these substances acted synergistically [93]. In the study by Quarta et al., it was shown that bromelain has the above-mentioned properties, but the combination with other substances (*Harpagophytum procumbens*, *Boswellia serrata*, *Curcuma longa*, aescin) gives better results. In the case of degenerative joint disease, it not only has an anti-inflammatory effect but also inhibits angiogenesis and osteophyte formation [94]. Other studies on the combination of bromelain with substances such as curcumin and hookvine worked well in vitro on OA. Bromelain alone, on the other hand, showed no effect [95].

The immunomodulatory effect of bromelain is also largely due to the deletion of CD7, CD8α, CD14, CD16, CD21, CD41, CD42a, CD44, CD45RA, CD48, CD57, CD62L, CD128a, and CD128b molecules from the surface of immune cells, which is associated with the functional silencing of immune cells. CD4, CD40, CD56, CD61, CD79, and CD132 were partially sensitive. It is worth noting that in this study the reactivity of CD5, CD11b, CD11c, CD13, CD15, CD18, and CD53 molecules increased, probably due to epitope exposure by proteolysis [48]. Secor et al. also showed that Br (EC 3.4.22.32), at a dose of 25–100 µg/mL, reduces both surface and soluble forms of CD25 on activated CD4+ T lymphocytes. The effect is dependent on cell activation and proteolytic activity of the enzyme [96].

The main effects of bromelain in the context of anti-inflammatory and immunomodulatory effects are summarized in Table 3.

### 3.3. Cardiovascular Diseases, Dyslipidemia, and Hypertension

One of the most common consequences of obesity is cardiovascular disease associated with dyslipidemia and hypertension. The effect of bromelain has been explored in this aspect. Ley et al. studied 68 Chinese patients with diabetes in a randomized, placebo-controlled, double-blind, and parallel design. Half took a bromelain supplement and the other half a placebo supplement with starch. The study demonstrated that the effects observed in human studies appear less pronounced than those in animal studies. The level of low-density lipoprotein cholesterol (LDL-C) and postprandial glycemia decreased, while the remaining lipoprotein fractions and CRP, glycated hemoglobin (Hgb1c), and fasting glucose did not change [75].

In the studies by Sulumer et al., rats exposed to bromelain with food had lower levels of total cholesterol (TC), triglycerides (TGs), and LDL-C despite tyloxapol-induced hyperlipidemia [88]. Atherosclerosis-prone apolipoprotein E-deficient (Apoe^−/−^) mice were fed a regular chow diet. Half of the mice received bromelain at 20 mg/kg body weight daily. After 4 weeks, parameters of atherosclerosis were examined. Levels of total cholesterol, non-HDL-c and triglycerides in plasma were lower in apoe^−/−^ mice treated with bromelain. In this group, aortic inflammation decreased, which may contribute to delayed atherosclerosis progression. Supplementation did not appear to affect mean arterial pressure [54]. In another study on an animal model, an HFD was used for 12 weeks. In the group with bromelain-enriched food, the level of total cholesterol was lower by about 15% and triglycerides by about 25% [77]. Table 4 summarizes studies on the effects of bromelain in the most common obesity-related diseases associated with low-grade inflammation.

### 3.4. Blood Coagulation and Fibrinolysis

Bromelain has been reported to influence blood coagulation by potentially increasing the fibrinolytic capacity of serum and inhibiting the synthesis of fibrin, a protein involved in blood coagulation. It may affect the conversion of plasminogen to plasmin, which causes fibrin degradation [99,100]. However, depending on the dose and time after administration, bromelain may also have an opposite prothrombotic effect. In a study, very low concentrations of bromelain injected intraperitoneally in rats (1.5 and 3 mg, compared with the previous study of 30 mg) showed an effect of increasing coagulation via platelet activation [101]. The route of administration may also be the cause of this paradox. Bromelain, as a protease, when injected intraperitoneally may cause inflammation through the digestive action of the peritoneal tissues, which may lead to platelet activation due to the action of tissue factor [101].

In the study by Eckert et al., blood coagulation parameters did not change in 16 patients with breast cancer compared to healthy individuals after 10 days of daily administration of 3000 FIP units of bromelain, except for the extension of activated partial thromboplastin time (APPT) from 38 to 46 s [102].

In animal studies, bromelain administered to rabbits lengthens prothrombin time from 80% to 250%, increases antiprothrombin time, and also elevates serum plasmin level. The minimum effective dose was at least 5 mg/kg [103]. In another study in rats, intravenous injection of bromelain at different doses changed the tested parameters. Compared with the control group, prothrombin time (PT), prothrombin levels, Factor X (Stuart-Factor), and fibrinogen levels in plasma were lowered. The results were dose-dependent but not statistically significant [104]. Bromelain injected intraperitoneally in rats also appears to reduce myocardial infarction extension, increase aortic flow, and improve recovery of left ventricular function during reperfusion compared with the control group [105]. Other studies suggest a potential antithrombotic effect both after oral and intravenous administration [106,107]. In a study on a mouse model on an HFD, which is supposed to reflect the nutritional conditions of subjects exposed to cardiovascular diseases, inhibition of the activation of the coagulation cascade and reduction in clot stability were demonstrated. Additionally, bromelain showed fibrinolytic activity [108]. It is worth mentioning the study by Kaur et al. [101], where mice were intraperitoneally injected with physiological saline or a clinically relevant dose of bromelain: 1.5 or 3 mg/kg. The total volume injected to each animal was 200 mL. After 20 min, blood samples were taken and coagulation was assessed. A paradoxical effect towards hypercoagulation was demonstrated; however, the compared data did not provide statistically significant conclusions. Additionally, human blood coagulation parameters were examined using thromboelastography (TEG) as well as conventional assays. Blood was collected from healthy individuals and from hypercoagulable individuals. The prepared sample was incubated with 0.4 U/mL bromelain, which caused PT prolongation by 47 and 22% and APTT prolongation by 20 and 10% in normal and hypercoagulable samples, respectively, and inhibited adenosine diphosphate (ADP)-induced platelet aggregation by 19% [101]. This study suggests complexity in the mechanisms responsible for bromelain interactions with the blood coagulation system.

Additionally, the results of Errasti et al. indicate differences in the effect of bromelain depending on the dose; at low concentrations it showed a procoagulant effect and at high concentrations an anticoagulant effect [99]. Studies on fibrinogen (FIB) and the pool of platelet-poor plasma from healthy donors showed an increased fibrinolytic effect and prolongation of the relative time of PT and APTT [100]. This effect has been confirmed in studies on normal and hypercoagulable blood samples [101,106]. Table 5 presents studies related to the action of bromelain on blood coagulation and fibrinolysis.

### 3.5. Nonalcoholic Fatty Liver Disease and Diabetes

Bromelain may exert a potential protective effect on the liver against a high-fat diet. In studies on the effect of bromelain on NAFLD, parameters of insulin sensitivity were described because the accumulation of fat in the liver is associated with impaired insulin action in the production of glucose and FFAs in serum. Increased FFAs cause peripheral and hepatic insulin resistance [109]. These reports were supported by findings from another randomized, placebo-controlled, double-blind, parallel efficacy study conducted in China examining the effect of 12 weeks of bromelain (1050 mg/day) on plasma fibrinogen [75]. Ley et al., in the study described in the previous subsection among diabetic patients, in the group taking the supplement, found no changes in Hgb1c and fasting glucose levels [75]. However, another study showed that bromelain supplementation may significantly improve insulin sensitivity [50].

C57BL/6 mice were fed an HFD and given bromelain for 12 weeks. Bromelain treatment reduced the accumulation of lipids in the liver compared to the control group without supplementation, which may suggest the protective effect of bromelain on the liver in individuals who use the Western-style diet [77]. Table 6 summarizes studies on the effects of bromelain in NAFLD and diabetes.

## 4. Pharmacological Properties, Bioavailability, and Aspects of Use

### 4.1. Pharmacological Properties

Bromelain exhibits a wide spectrum of pharmacological properties that are particularly relevant to metabolic and inflammatory disorders. At the molecular level, bromelain modulates adipocyte biology by influencing differentiation, apoptosis, and lipolysis through several signaling pathways. It has been shown to reduce the expression of C/EBPα and PPARγ mRNA, two transcription factors central to adipogenesis, thereby limiting preadipocyte differentiation [43]. In addition, bromelain may interfere with the Akt–TSC2–mTORC1 pathway, leading to inhibition of PPARγ target genes involved in lipid metabolism, such as ap2 (FABP4), FAS, LPL, CD36, and ACC [43,44].

As part of its pharmacological action on adipose tissue, bromelain induces apoptosis of mature adipocytes, partly via modulation of Akt phosphorylation and TNF-α-dependent pathways, while sparing preadipocytes [45]. Through its effect on TNF-α and IRS-1 phosphorylation, bromelain may alleviate insulin resistance by restoring GLUT4 function and glucose uptake [46,47].

Beyond direct effects on adipocytes, bromelain displays immunopharmacological activity in adipose tissue and other metabolic organs. It reduces infiltration of pro-inflammatory M1 macrophages and promotes their polarization toward an anti-inflammatory M2 phenotype, thereby improving metabolic homeostasis [48]. Furthermore, bromelain attenuates metabolic endotoxemia by reducing lipopolysaccharide (LPS) translocation from the gut into the bloodstream, preventing TLR4-driven cytokine production (TNF-α, IL-6, IL-1β) and impairment of insulin signaling [51,52]. Its anti-inflammatory pharmacological profile includes inhibition of NF-κB activation, downregulation of adhesion molecules (ICAM-1, VCAM-1), and suppression of MAPK, COX-2, PGE2, and AP-1 transcription factors [53,54,55,56,57,58]. Bromelain also reduces chemokines (MIP-1α, MIP-1β, MCP-1) [59,60,61] and iNOS expression, thereby lowering oxidative stress [62]. In vitro studies additionally show that bromelain can remove certain immune cell surface receptors (e.g., CD128a/b), limit neutrophil migration, and attenuate immune cell activation [63,64].

Taken together, these pharmacological effects position bromelain as a compound with potential to counteract obesity-associated low-grade inflammation by downregulating pro-inflammatory cytokines (TNF-α, IL-1β, IL-6) [66], supporting antioxidant defense mechanisms [66], and modulating gut-derived inflammatory triggers such as LPS [51,52,67]

### 4.2. Clinical Applications and Therapeutic Use

Bromelain may have potential properties that support weight loss and anti-inflammatory effects, and may reduce the metabolic consequences of both situations. Considering only anthropometric parameters such as body weight, BMI, WC, and WHR, a dose of 1000–1050 mg/day for 8–12 weeks has been reported to be effective [52,60]. Two daily doses of 500 mg bromelain administered orally for 8 weeks reduced leptin and pro-inflammatory cytokine levels [43]. It is assumed that 250–2000 mg/day (500–5000 FIP units) of bromelain divided into 2–3 daily doses has a preventive effect on metabolic diseases [110]. In patients with diabetes, parameters such as HOMA-IR or fasting glucose were improved by a bromelain dose of 1000–1050 mg/day in 2–3 doses [50,68]. Similar doses lowered blood pressure and improved the lipid profile after 12 weeks of supplementation [75]. In addition, other aspects of bromelain use can be considered. Its administration before surgery may facilitate recovery and reduce postoperative inflammation and pain [111]. Therapeutic effects have been reported at doses as low as 160 mg/day, with higher doses (≥750 mg/day) often associated with greater efficacy. It is generally recommended to take bromelain at least 1 h before a meal. The tablets must be coated in such a way that they are resistant to digestion in the stomach [112].

### 4.3. Dosage, Pharmacokinetics, and Safety

Bromelain is a proteolytic enzyme mixture that is sensitive to low pH and gastric proteolysis. It is generally recommended to take bromelain at least 1 h before a meal since the tablets are coated in a way that makes them resistant to digestion in the stomach [112]. The gastrointestinal absorption of bromelain after oral administration is 40%, while its plasma half-life is approximately 6–9 h [112,113]. The therapeutic dosage of bromelain in the adult population is generally considered to be 250–2000 mg [110]. Serum concentrations after standard doses (e.g., 1–2 g) are very low (<1 µg/mL), which is significantly lower than the effective concentrations in vitro (10–100 µg/mL) [43,112]. A total of 500 milligrams of bromelain per kilogram of body weight per day administered orally to rats did not cause any changes in food intake; growth; histology of the heart, kidneys, and spleen; or in hematological parameters [114]. When doses of 1500 mg/kg per day were administered to rats, no significant adverse effects were observed. Studies on dogs have shown that doses of even 750 mg/day for half a year did not lead to any toxic effects [114]. In an analysis of 12 placebo-controlled studies, only one reported side effects such as diarrhea, nausea, occasional stomach upset, and allergic reactions, which were observed in only 1.8% of participants. Only isolated reports of mild adverse reactions, such as rash or hives, have been documented by companies producing bromelain supplements [115]. However, some sources state that due to its anticoagulant properties, bromelain should not be used in subjects with reduced blood clotting capacity or two to three weeks before dental or surgical intervention.

### 4.4. Contraindications and Drug Interactions

Bromelain supplementation is not recommended for pregnant women and childbirth. People with liver and kidney disease should only supplement bromelain with a doctor’s advice [116]. It is worth considering possible interactions of bromelain supplementation with medications. The listed medications that may interact with this substance are antiplatelet or anticoagulant medications, including aspirin, heparin, warfarin, and clopidogrel, as well as nonsteroidal anti-inflammatory drugs (NSAIDs) such as ibuprofen and naproxen. It should only be used under the supervision of a practitioner [116].

### 4.5. Limitations of Recent Studies and Strength of Current Evidence

There are many doubts in the research on the effectiveness of bromelain. Bromelain interventions in numerous experiments presented in this review differed significantly in dose, method, model, and duration. The observations were also diverse. Human studies are often conducted on small groups with high heterogeneity, which significantly differ in health status, age, diet, or lifestyle, which disturbs the observations. The enzymes used in the experiments come from different extraction methods (from stem and fruits), which can affect the enzyme content and pharmacological activity [53]. All these aspects make it impossible to draw direct conclusions about the effect. Further difficulties are provided by translating in vitro or animal model doses into supplementation in humans in order to ensure efficacy and safety. It should also be emphasized that in future studies, standardized, purified preparations with specific units of activity and exposure measurements should be used. The need to study the use of bromelain in the long term is also questionable, because even natural compounds with long-term use can potentially increase side effects [117].

## 5. Materials and Methods

This review article was created after analyzing data obtained from PubMed, Scopus, and Google Scholar databases. The search was conducted from 1 February 2025, to 1 May 2025. The following search terms were used: “bromelain” or “bromelain” and one of the following: “obesity”, “inflammation”, “cardiovascular diseases”, “metabolic disease”, “diabetes”, “insulin resistance”, “arterial hypertension”, “dyslipidemia”, “hypercholesterolemia”, and “medicine”. Only English-language articles were included. No publication date restrictions were applied to ensure comprehensive coverage of the topic. Articles were selected based on their relevance to bromelain’s mechanisms, clinical applications, or pharmacological effects in metabolic and inflammatory disorders.

## 6. Conclusions

With the growth of the worldwide obesity epidemic, as well as the overuse of some drugs available for the treatment of obesity, there is an urgent need to further establish the role of phytotherapeutic compounds in the treatment of obesity. Bromelain may have potential benefits in obesity management by influencing adipogenesis, lipolysis, adipocyte apoptosis, anti-inflammatory, and antioxidant properties. Additionally, bromelain has been associated with improved insulin sensitivity. However, it should be noted that there are a lack of clinical trials in humans related to its weight loss effect. There is a high need for randomized clinical trials in obese people without additional diseases. However, in light of current research, bromelain supplementation may be considered a potential adjunctive strategy to support other pharmacological methods, as it appears to act synergistically with many substances. It is noteworthy that the generally low frequency of reported side effects associated with supplementation, even at high doses, suggests that it may represent a compound with potential therapeutic properties. Future studies should also investigate the potential effects of bromelain on the gut microbiota and the circadian rhythm of adipose tissue.

## Figures and Tables

**Table 1:** Summary of major consequences of obesity.

Associated Diseases	Ref.	Findings Correlating with Obesity
Insulin resistance (IR)	[22,23,24]	Insulin resistance serves as a pivotal link between development of NAFLD and consequent progression of atherosclerotic cardiovascular diseases in obese patients with increased levels of leptin, lipotoxicity, and mitochondrial dysfunction and decreased levels of adiponectin being the underlying mechanisms. Insulin resistance along with oxidative stress, inflammation, gut dysbiosis, renin–angiotensin–aldosterone system (RAAS) overactivity, and endothelial dysfunction have been stated as different mechanisms that collectively associate the pathophysiology between NAFLD and CVD with obesity being its primary driver.
Type 2 Diabetes Mellitus (T2DM)	[25]	Abdominal obesity increases the risk of glucose intolerance and insulin resistance, which are key factors in the development of type 2 diabetes. Studies have demonstrated a steep increase in the relative risk of developing type 2 diabetes with increasing body mass index (BMI). The higher levels of free fatty acids (FFAs) in obesity impair glucose use and increase hepatic glucose output, contributing to the development of T2DM.
Cardiovascular Disease (CVD)	[19,21,25]	Obesity is widely recognized as an independent risk factor for CVD, including coronary heart disease and heart failure. Mechanisms linking obesity to CVD include increased vascular volume, sympathetic nervous system activation, and changes in Na^+^/H^+^-ATPase activity, all potentially influenced by hyperinsulinemia. Elevated BMI values were responsible for 4 million deaths in 2015, with two-thirds of this number attributed to cardiovascular disease. Numerous studies have identified obesity as an important risk factor for the development of heart failure.
Nonalcoholic Fatty Liver Disease (NAFLD)	[19,22]	Increased FFAs in obesity contribute to lipid accumulation in the liver, leading to inflammation and liver damage. Studies have shown that weight loss can improve liver function and reduce fat accumulation in individuals with NAFLD.
Hypertension	[22,26,27]	Multiple studies state the activation of RAAS due to obesity by upregulation of angiotensinogen, angiotensin 1, and angiotensin-converting enzyme to be the cause of developing hypertension. Obesity predisposes renal sodium reabsorption thereby necessitating an increase in arterial pressure to maintain sodium balance. Other potential mechanisms include a decrease in natriuretic peptides with subsequent impairment in salt excretion.
Stroke	[28,29,30]	Obesity-related chronic inflammation plays an essential role in the progressive elevation of stroke risk, as increased plasma levels of interleukin-6 (IL-6) and C-reactive protein (CRP) are well-established inflammatory biomarkers implicated in the pathogenesis of cerebrovascular events. Although the obesity paradox implies obesity to have a protective stroke outcome in patients, such as reduced risk of recurrent stroke and improved functional recovery, its existence has not been confirmed by all studies. In spite of this, clinical evidence in an experimental study of obesity in rat models showed increased ischemic brain damage in the obese rodents.
Chronic Kidney Disease (CKD) and Other Renal Complications	[31,32,33,34]	Various population-based studies have shown direct link between high BMI in obese patients and progressive loss in GFR over time and incidence of end-stage renal disease (ESRD). Cardiovascular and metabolic effects such as high blood pressure and diabetes mellitus due to obesity aid in the disruption of kidney function. With visceral adipose tissue being the primary source of cytokine release affecting renal hemodynamics, persistent inflammation also contributes to fibrosis in the kidney. Furthermore, a cohort analysis found that participants with *stage 2* hypertension had a 3.4-fold-higher chance of acquiring glomerulonephritis-related ESRD and a 2.2-fold-higher risk of developing non-glomerulonephritis-related ESRD than those with normal blood pressure.
Fertility	[35,36]	Ovulatory dysfunction has been noted to be more common in women with obesity; in addition, greater BMI at the age of 18 years predicted anovulatory infertility with or without polycystic ovary syndrome (PCOS), which is a common symptom of obesity. Recent evidence states sperm epigenetics are altered in men with obesity. Moreover, increased abdominal adiposity in men of subfertile couples has been associated with reduced sperm count, concentration, and motility.
Cancers	[37,38,39,40]	Systemically, metabolic syndrome, including dyslipidemia and insulin resistance, occurs in the setting of adipose inflammation and operates in concert with local mechanisms to sustain the inflamed microenvironment and promote tumor growth. Recent investigations have established a significant association between overweight or obesity, defined as a BMI of 25 or greater, and the increased risk of developing at least thirteen distinct malignancies. These include cancers of the esophagus, stomach, colon and rectum, liver, gallbladder, pancreas, postmenopausal breast, uterus, ovary, kidney, meningioma, thyroid, and multiple myeloma. Dysregulation of adipocytes and infiltrating immune cells in the adipose tissue results in chronic inflammation, which plays a crucial role in the progression of obesity-associated cancers.

T2DM—type 2 diabetes mellitus, BMI—body mass index, FFAs—free fatty acids, CVD—cardiovascular diseases, NAFLD—non-alcoholic fatty liver disease, IR—insulin resistance, RAAS—renin–angiotensin–aldosterone system, CKD—chronic kidney disease, ESRD—end-stage renal disease, IL6—interleukin 6, CRP—c-reactive protein, PCOS—polycystic ovary syndrome.

**Table 2:** Bromelain effects, in vitro (Cell) and in vivo (Human, Animal), on obesity.

Fields of Study	Ref.	Dosage	Subjects	Outcomes
Human	[50]	1000 mg/d (2 × 500 mg) for 8 weeks, p.o.;	obese diabetic patients	↓ BMI, waist circumference (WC), waist-to-hip ratio (WHR), ↓ serum leptin,
	[74]	1050 mg/d (3 × 350 mg) for 12 weeks, p.o.	diabetic patients with normal weight	n.c. body mass, hip circumference (HC), and WC
Animal	[53]	20 mg/kg/day for 4 weeks, p.o.	Apoe^−/−^ mice with hyperlipidemia (apolipoprotein E-deficient mouse model of atherosclerosis)	↓ body mass, WAT weight, ↑ BAT weight, ↓ size of droplet of BAT and WAT
	[75]	100 µL high-internal-phase oil-in-water emulsions (HIPEs) with added bromelain fibrils for 10 weeks, p.o.	mice +HFD	↑ excretion of fats in the feces
	[76]	20 mg/kg/day for 12 weeks, p.o.	C57BL/6 mice + HFD	↓ body weight by ~30%, ↓ organ weight by ~20% in liver weight and ~40% in white adipose tissue weight
Cell	[43]	Enzyme Commission Number (EC) 3.4.22.32, 10–100 µg/mL	preadipose 3T3 clonal cell line	↓ adipogenesis, ↑ apoptosis, ↑ lipolysis

The effects of bromelain are marked as follows: ↑ increased, ↓ decreased, n.c.—no change; method of administration: p.o.—orally (per os), Apoe^−/−^ mice—atherosclerosis-prone apolipoprotein E-deficient mice, BAT—brown adipose tissue, BMI—body mass index, HC—hip circumference, HFD—high-fat diet, WC—waist circumference, WAT—white adipose tissue.

**Table 3:** Bromelain anti-inflammatory and immunomodulation effects, in vitro (Cell) and in vivo (Human, Animal).

Fields of Study	Ref.	Dosage	Subjects	Outcomes
Human	[79]	1000 mg/d (2 × 500 mg) for 8 weeks, p.o.	obese diabetic patients	↓ leptin, IL-6, TNF-α
Animal	[82]	EC 3.4.22.32, 35–200 µg, i.v.	BALB/c + sheep red blood cells (SRBCs) mice	↑ B cell ↓ mRNA IL-2
	[83]	EC 3.4.22.32, 1000 mg/kg/day for 3 weeks, p.o.	Wistar albino rats	↓ TNF-α, IL-10, MCP-1, PGE2, ↓ NF-κB, CRP, MPO
	[84]	EC 3.4.22.33,10, 80 mg/kg/day for 14 days, p.o.	Sprague-Dawley rats + 2,4,6-trinitrobenzene sulfonic acid (TNBS)	↑ occluding, ↓ TNFRs, NF-κB, MLCK, FD-4,
	[86]	EC 3.4.22.32, 5 mg/kg/d, or 10 mg/kg/d, p.o. for 14 days	Wistar albino rats	↓ TNF-α, IL-6, M-CSF, MMP-8, RANKL, ↑ OPG
	[87]	EC 3.4.22.32, 30 mg/kg and 50 mg/kg, p.o.	Wistar albino rats	↓ IL-1β, IL-6, TNF-α, PGE2, NF-κB, iNOS, NO3-/NO2-, glutamine
	[88]	250 mg/kg/day, p.o.;	rats	↓ IL-1β, IL-6, TNF-α
	[97]	10 and 20 mg/kg, p.o.	rats	↓ P, PGE2
Cells	[56]	6.25–5000 µg/mL	RAW264.7—mouse macrophages +LPS	↓ iNOS, COX-2 (dose-dependent), ↓ MAPK (p-ERK, p-JNK, p-p38), ↓ c-Fos expression and c-Jun phosphorylation, ↓ NO (dose-dependent)
	[55]	EC 3.4.22.32, 2.5, 5, 10, 20 µg/mL	human dental pulp cells (hDPCs) + LPS	↓ IL-1β, IL-6, IL-8 (mRNA + protein), ↓ ICAM-1, VCAM-1 ↓ ERK, p38 MAPK, NF-κB (p65) n.c. JNK
	[59]	EC 3.4.22.32, 1 mg/mL	colonic mucosa biopsies from patients with Crohn’s disease or ulcerative colitis	↓ G-CSF, GM-CSF, IFN-γ, CCL4, MIP-1β, TNF-α
	[61]	35 mg/L (0.035 mg/mL)	stomach, intestinal, and chondrocyte human cellular models (AGS, Caco-2, and SW1353)	↓ IL-8, COX-2, iNOS
	[64]	EC 3.4.22.32, from 1 µg/mL to >1000 µg/mL	human leukocytes in whole blood, PBMCs	↓ CD7, CD8α, CD14, CD16, CD21, CD41, CD42a, CD44, CD45RA, CD48, CD57, CD62L, CD128a, CD128b, CD4, CD40, CD56, CD61, CD79, CD132. ↑ CD5, CD11b, CD11c, CD13, CD15, CD18, CD53
	[65]	EC 3.4.22.32, 2.5, 5, 10, 20, 40 µg/mL	human gingival fibroblasts (HGFs) + AGEs	↓ IL-6, IL-8, p16, ↓ NF-kB, MAPK/ERK
	[67]	E.C. 3.4.22.32, 50–100 μg/mL	PBMCs and monocytic leukemia THP-1 cells + LPS	↓ TNF-α, IL-1β, IL-6
	[80]	10–1000 µg/mL	PBMCs obtained from subjects with encephalomyelitis disseminate and healthy control group	↑IL-6, GM-CSF, TNF-α, IFN-γ
	[81]	5.4 FIP-E./mg	modified mixed lymphocyte culture (MMLC)	↑IL-6,
	[82]	EC 3.4.22.32, 0–200 µg/mL	CD41 T cells, CD81 T cells, B cells from mouse splenocytes	↑ proliferation (in splenocytes), ↓ IL-2 ↑costimulation by B cells
	[84]	EC 3.4.22.33 <80 µg/mL	intestinal epithelial cells IEC-6 (rat) and Caco-2 (human) + LPS	↓ TNFR2, NF-κB, MLCK ↑ TER
	[85]	EC 3.4.22.32, 10 mg/mL in nanocomposites and 500 µg/mL free bromelain	Caco-2 (intestinal line), PBMCs (blood mononuclear lymphocytes) celiac and healthy + gliadin	↓ CCR5, CXCR3 ↓ IL-1β, IL-6, TNF-α, IFN-γ ↑ IL-10, cytotoxic T lymphocyte antigen 4 (CTLA-4)
	[89]	EC 3.4.22.32, 100 µg/mL	human monocytic cell line U937 + LPS	↓ IL-1β, IL-6, IL-8, COX-2, MIP-1α/β, MCP-1
	[90]	EC 3.4.22.3210–40 µg/mL	SW982 synovial fibroblasts + TNF-α	↓ TNF-α, IL-1β, IL-6, IL-8, ↓ phosphorylation NF-κB p65, IκB-α, ↓ activation MAPK: p38, JNK n.c. ERK
	[91]	crude (CBM) and purified rhizome bromelain (PBM) (CBM: 20, 40 and 80 µg/mL) (PBM: 10, 20 and 40 µg/mL)	RAW 264.7 macrophage cells + LPS	CBM: ↓ IL-6, NO (only with 80 µg/mL), p38, iNOS and COX-2 n.c. TNF-α, ERK1/2, JNK, p65, IκB-α. PBM: ↓ IL-6, TNF-α, NO, ERK1/2, JNK, p38, p65, IκB-α., iNOS and COX-2
	[92]	EC 3.4.22.3215 100 mg	PBMCs obtained from healthy subjects	↓ TNF-α, IL-1β, IL-6
	[93]	EC 3.4.22.32, 20 ng/mL, after 3 and 7 days	mesenchymal stem cells (MSCs)	↑ IL-10 ↓ IL-1 n.c. IL-2, IL-6 and IL-8
	[94]	EC 3.4.22.32, 50–100 µg/mL	human monocytoid THP-1 cells, (Human Microvascular Endothelial Cells-1 (HMEC-1)	↓ IL-6, IL-8, MMP-9, n.c. COX-2
	[95]	EC 3.4.22.32, 14.7 µg/mL	human synovium from OA patients + LPS	n.c. IL-6, NGF, MMP-1, MMP-3, MMP-13, PGE2, MMPs
	[96]	EC 3.4.22.32, 25–100 µg/mL	CD4+ CD25 cells isolated from mouse spleens	↓ CD25 and sCD25
	[98]	EC 3.4.22.32, 10–100 µg/mL	RAW264.7—mouse macrophages (ATCC)	↓ NF-κB, NO

The effects of bromelain are marked as follows: ↑ increased, ↓ decreased, n.c.—no change; method of administration: p.o.—orally; i.v.—intravenously (intravenosa); AGEs—advanced glycation end products; CD—cluster of differentiation; COX-2—cyklooksygenaza-2; CRP— C-reactive protein, CTLA-4— cytotoxic T lymphocyte antigen 4; ERK, JNK, MAPK, p38—protein kinases involved in cellular signaling pathways; FD 4—fluorescein isothiocyanate-dextran 4; hDPCs—human dental pulp cells; HGFs—human gingival fibroblasts; IL-1β, IL-2, IL-6, IL-8, IL-10—interleukine (different types); iNOS—inducible nitric oxide synthase; LPS—lipopolysaccharide; M-CSF—macrophage colony-stimulating factor; MCP-1—monocyte chemoattractant protein-1; MLCK—myosin light chain kinase; MMP-1, MMP-3, MMP-8, MMP-9, MMP-13—matrix metalloproteinases; NF-κB—nuclear factor kappa B; NO—nitric oxide; OA—osteoarthritis; OPG—osteoprotegerin; PBMC—peripheral blood mononuclear cell; PGE2—prostaglandin E2; RANKL—receptor activator of nuclear factor κ B ligand; SRBC—sheep red blood cell; TER—transepithelial electrical resistance; TNF-α—tumor necrosis factor alpha.

**Table 4:** Bromelain effects in vivo (Human, Animal) on hypercholesterolemia and heart disease.

Fields of Study	Ref.	Dosage	Subjects	Outcomes
Humans	[75]	1050 mg/d (3×350 mg) for 12 weeks, p.o.	diabetic patients with normal weight	n.c. blood pressure; n.c. total cholesterol, TG, high-density lipoprotein cholesterol (HDL-C); ↓ LDL
Animals	[54]	20 mg/kg/day, for 4 weeks, p.o.	Apoe^−/−^ mice with hyperlipidemia	n.c. mean arterial pressure; ↓ TC, TG, no-HDL-C; ↓ inflammation of the aorta, formation of atherosclerosis
	[77]	20 mg/kg/day for 12 weeks, p.o.	C57BL/6 mice + HFD	↓ TC ~15% ↓ TG ~25%
	[88]	250 mg/kg/day, p.o.	rats	↓ TC, TG, LDL-C

The effects of bromelain are marked as follows: ↑ increased, ↓ decreased, n.c.—no change; method of administration: p.o.—orally (per os); Apoe^−/−^ mice—atherosclerosis-prone apolipoprotein E-deficient mice; HDL-c—high-density lipoprotein cholesterol; HFD—high-fat diet; LDL-c—low-density lipoprotein cholesterol; TC—total cholesterol; TG—triglyceride.

**Table 5:** Bromelain effects, in vitro (Cell) and in vivo (Human, Animal), on blood coagulation and fibrinolysis.

Fields of Study	Ref.	Dosage	Subjects	Outcomes
Human	[102]	3000 Federation Internationale Pharmaceutique units (F.I.P units) for 10 days, p.o.	breast cancer patients and healthy subjects	↑ activated partial thromboplastin time was increased from 38 to 46 s n.c. leaving prothrombin time and plasminogen
Animal	[103]	minimum 5 mg/kg/day, p.o.	rabbits	↑ antiprothrombin time and serum plasmin
	[104]	1, 5, 10, 20, and 30 mg/kg, i.v.	rats	↓prothrombin time, prothrombin levels, FactorX (Stuart-Factor), fibrinogen levels
	[105]	10 mg/kg twice a day for 15 days, i.p.	Sprague-Dawley rats	↓ infarction spread ↑aortic flow, ↑left ventricular functional recovery throughout reperfusion
	[106]	(a) 60 mg/kg, p.o.; (b) 30 mg/kg, i.v.	rats	(a) ↓ thrombus formation in 11% of arterioles and 6% of venules (b) ↓ thrombus formation in arterioles (13%) and venules (5%)
	[107]	25 and 100 mg/kg, p.o.	rats	↑ serum fibrinolytic activity ↓absorption time of hematoma ↓ pentobarbital-induced sleeping time
	[108]	20 mg/kg/day for 12 weeks, p.o.	C57BL/6 mice + HFD	↓ PT, APTT, and FIB (fibrinogen)
	[101]	1.5 mg/kg, i.p.	CD1 mice (mice type CD1)	↑ hypercoagulation
Cell	[99]	EC 3.4.22.32, (B4882), from 0.003 casein units per milliliter (CU/mL) to 10 CU/mL	(a) fibrinogen; (b) platelet-poor plasma of healthy donors	(a) ↑ fibrinolytic effect (b)↑ PT, APTT
	[100]	EC 3.4.22.32, 0.8 μL at a concentration of 1.0 UC/mL	(a) fibrinogen; (b) pool of platelet-poor plasma	(a)↑ fibrinolytic effect (b)↑ PT, APTT
	[101]	0.4 U/mL	whole blood samples taken from healthy individuals and hypercoagulable	↑ PT by 47% (healthy) and 22% (hypercoagulable) ↑ APTT by 20% (healthy) and 10% (hypercoagulable) ↓ platelet aggregation
	[106]	10 mg/mL	platelets with thrombin (0.2 U/mL).	↓ platelet aggregation

The effects of bromelain are marked as follows: ↑ increased, ↓ decreased, n.c.—no change; method of administration: p.o.—orally (per os); i.v.—intravenously (intravenosa); i.p.—intraperitoneally (intraperitoneal); FIB—fibrinogen; APTT—activated partial thromboplastin time; FIP unit—Federation Internationale Pharmaceutique unit; PT—prothrombin time.

**Table 6:** Bromelain effects in vivo (Human, Animal) on NAFLD and diabetes.

Fields of Study	Ref.	Dosage	Subjects	Outcomes
Human	[50]	1000 mg/d (2 × 500 mg) for 8 weeks, p.o.	obese diabetic patients	↓Homeostasis Model Assessment of Insulin Resistance (HOMA-IR) n.c. fasting glucose
	[75]	1050 mg/d (3 × 350 mg) for 12 weeks, p.o.	diabetic patients with normal weight	↓post prandial blood glucose ↓ fasting glucose, n.c. Hgb1c
Animal	[77]	20 mg/kg/day for 12 weeks, p.o.	C57BL/6 mice + HFD	↓ lipid accumulation in the liver

The effects of bromelain are marked as follows: ↑ increased, ↓ decreased, n.c.—no change; method of administration: p.o.—orally (per os); Hgb1c—glycated hemoglobin; HFD—high-fat diet; HOMA-IR—Homeostasis Model Assessment of Insulin Resistance.

## Data Availability

Not applicable.

## Abbreviations

The following abbreviations are used in this manuscript:

3T3-L1	Preadipocyte cell line derived from mouse embryonic fibroblasts
ACC	Acetyl-CoA carboxylase
AGEs	Advanced glycation end-products
Akt	Protein kinase B (PKB), also known as Akt
AP-1	Activator protein 1 (transcription factor complex including c-Jun and c-Fos)
Ap2	Adipocyte protein 2 (also known as FABP4—fatty acid binding protein 4)
Apoe^−/−^ mice	Apolipoprotein E-deficient mice (model of atherosclerosis)
APTT	Activated partial thromboplastin time
BAT	Brown adipose tissue
BMI	Body mass index
C/EBP	CCAAT/enhancer-binding protein
CCI	Chronic Constriction Injury
CD	Cluster of differentiation
CD (np. CD7, CD8α, CD14 itd.)	Cluster of differentiation
CD1 mice	Nice-type CD1
CD128a/CXCR1	CXC chemokine receptor 1
CD128b/CXCR2	CXC chemokine receptor 2
CKD	Chronic kidney disease
COX-2	Cycloxygenase
CPT-1	Carnitine palmitoyltransferase 1
CRP	C-reactive protein
CTLA-4	Cytotoxic T lymphocyte antigen 4
CU/mL	Casein units per milliliter
CVD	Cardiovascular disease
EC	Enzyme Commission Number
EP	Leptin gene expression
ERK	Extracellular signal-regulated kinase
ERK, JNK, MAPK, p38	Protein kinases involved in cellular signaling pathways
ESRD	End-stage renal disease
FAS	Fatty acid synthase
FD 4	Fluorescein isothiocyanate-dextran 4
FFAs	Free fatty acids
FIB	Fibrinogen
FIP unit	Federation Internationale Pharmaceutique unit
Gia1	Inhibitory G protein alpha subunit 1
GLUT-2	Glucose transporter type 2
GLUT4	Glucose transporter type 4
HC	Hip circumference
HDL-C	High-density lipoprotein cholesterol
hDPCs	Human dental pulp cells
HFD	High-fat diet
Hgb1c	Glycated hemoglobin
HGFs	Human gingival fibroblasts
HIPEs	High internal phase emulsions
HOMA-IR	Homeostasis Model Assessment of Insulin Resistance
HSL	Hormone-sensitive lipase
IBD	Inflammatory bowel disease
ICAM-1	Intercellular adhesion molecule 1
IFN-γ	Interferon gamma
IL-1β	Interleukin 1 beta
IL-6	Interleukin 6
IL-8	Interleukin 8
IL-10	Interleukin 10
iNOS	Inducible nitric oxide synthase
IR	Insulin resistance
IRS-1	Insulin receptor substrate 1
JNK	C-Jun N-terminal kinase
LDL-C	Low-density lipoprotein cholesterol
LPL	Lipoprotein lipase
M1	Classically activated (pro-inflammatory) macrophages
M2	Alternatively activated (anti-inflammatory) macrophages
MAPK	Mitogen-activated protein kinase
MCL	Mitotic clonal expansion
MCP-1	Monocyte chemoattractant protein 1
M-CSF	Macrophage colony-stimulating factor
MIP-1α	Macrophage inflammatory protein 1 alpha
MIP-1β	Macrophage inflammatory protein 1 beta
MLCK	Myosin light chain kinase
MMP-8, MMP-1, MMP-3, MMP-13, MMP-9	Matrix metalloproteinases
mRNA	Messenger ribonucleic acid
mTORC1	Mechanistic target of rapamycin complex 1
NAFLD	Non-alcoholic fatty liver disease
NF-κB	Nuclear factor kappa B
NK	Natural killer cells
NO	Nitric oxide
OA	Osteoarthritis
OPG	Osteoprotegerin
p38	p38 MAP kinase (a subfamily of MAPK)
PBMC	Peripheral blood mononuclear cell
PCOS	Polycystic ovary syndrome
PDE3B	Phosphodiesterase 3B
PGE2	Prostaglandin E2
PPARγ (PPARc)	Peroxisome proliferator-activated receptor gamma
PT	Prothrombin time
RANKL	Receptor activator of nuclear factor κ B ligand
RAAS	Renin–angiotensin–aldosterone system
SBM	Stem bromelain
SRBC	Sheep red blood cells
SREBP-1c	Sterol regulatory element-binding protein 1c
T cells	T lymphocytes
T2DM	Type 2 diabetes mellitus
TC	Total cholesterol
TER	Transepithelial electrical resistance
TG	Triglyceride
TLR4	Toll-like receptor 4
TNF-α	Tumor necrosis factor alpha
TSC2	Tuberous sclerosis complex 2
UC/mL	Enzymatic activity unit concentration
VCAM-1	Vascular cell adhesion molecule 1
ICAM-1	Intercellular cell adhesion molecule 1
WAT	White adipose tissue
WC	Waist circumference
WHR	Waist-to-hip ratio
